# Differential changes in anatomical, molecular and functional determinants of intestinal glucose absorption during murine pregnancy

**DOI:** 10.14814/phy2.70493

**Published:** 2025-08-05

**Authors:** T. Sebastian Overduin, Georgia S. Clarke, Hui Li, Richard L. Young, Kathryn L. Gatford, Amanda J. Page

**Affiliations:** ^1^ School of Biomedicine The University of Adelaide Adelaide South Australia Australia; ^2^ Nutrition, Diabetes & Gut Health, Lifelong Health Theme, South Australian Health and Medical Research Institute Adelaide South Australia Australia; ^3^ Robinson Research Institute, The University of Adelaide Adelaide Australia; ^4^ Adelaide Medical School The University of Adelaide Adelaide South Australia Australia

**Keywords:** glucose, mouse, nutrient transport, pregnancy, small intestine

## Abstract

Pregnancy demands increased food intake and nutrient delivery to support the growing conceptus. The timing and regional specificity of adaptations in small intestinal (SI) anatomy and transport, and their functional consequences, remain unclear. We therefore assessed anatomy and expression of macronutrient transporters in the duodenum, jejunum, and ileum collected from time‐mated pregnant C57BL/6 mice at gestational day (GD) 6.5, 12.5, or 17.5 and age‐matched nonpregnant females. Ex vivo active glucose transport was measured in each SI region of nonpregnant and GD17.5 mice. The SI was 20% heavier (*p <* 0.001) and 10% longer (*p =* 0.027) and SI villi were 18% longer (*p <* 0.001) in late pregnant than nonpregnant mice. Differences in relative carbohydrate (*Slc5a1*, *Slc2a2*, and *Slc2a5*) and amino acid (*Slc6a19*) transporter expression between pregnancy stages were region‐specific, while expression of the fatty acid transporter *Fabp2* was lower in all pregnant groups. Despite anatomical and molecular changes in support of an increase in SI capacity for nutrient absorption, active glucose transport per unit area was similar in nonpregnant and late‐pregnant mice. Increased nutrient demand during pregnancy may therefore be met largely through SI expansion and slower nutrient transit, although contributions of other nutrient transport mechanisms require evaluation.

## 1 INTRODUCTION

The small intestine (SI) is the primary site of nutrient uptake in rodents and humans, and is highly plastic, able to respond to changes in food intake and nutrient demand. The SI is comprised of three distinct regions: the duodenum, jejunum, and ileum, each of which has unique anatomy, nutrient transporter expression, and function (Kiela & Ghishan, 2016; Volk & Lacy, 2017). Nutrient uptake from the SI lumen occurs by passive and facilitated transport, and the contribution of each varies between nutrients. In facilitated transport, nutrient‐specific transporters located at the apical and basolateral membranes of the absorptive cells (enterocytes) transport nutrients across the SI epithelium and into the maternal circulation (Kiela & Ghishan, 2016). Increased nutrient uptake capacity can thus be generated by overall expansion of the SI, increased villi length to increase the epithelial surface area, and/or upregulation of nutrient transporters.

Pregnancy demands adaptations in food intake and nutrient uptake to support the growth of the fetus and placenta and maternal tissue gain, including adipose deposition, which acts as an energy store to support lactation (López‐Luna et al., 1991). To meet these demands, food intake increases during pregnancy by 10%–15% in humans and 25%–50% in rodents (Clarke et al., 2021). In concert, the SI undergoes anatomical, molecular, and functional adaptations during pregnancy which increase its capacity for nutrient uptake and delivery (Overduin et al., 2024). These include slowed transit of food through the gastrointestinal tract in the later stages of pregnancy, particularly the intestine, with unchanged gastric emptying (Overduin et al., 2024). Although circulation volumes increase substantially, by ~50% during human pregnancy (Soma‐Pillay et al., 2016), there is limited evidence for increases in blood supply to the SI (Overduin et al., 2024), with portal vein blood flow stable across pregnancy stages in a longitudinal study in humans (Clapp 3rd et al., 2000), and similar in pregnant and nonpregnant rabbits (Pere et al., 1992). In rats and mice, increases of up to 80% in SI weight and length by late pregnancy have been reported, although these increases are generally smaller than those seen during lactation (Overduin et al., 2024). There is also some, although variable, evidence for SI microstructural changes in pregnant rats, including longer jejunal villi at mid‐ and late‐pregnancy, but inconsistent effects of pregnancy on duodenal and ileal villi length (Overduin et al., 2024). In contrast, limited data from mice and shrews do not suggest elongation of villi during pregnancy (Overduin et al., 2024). Transcript and protein expression of many micronutrient transporters, and intestinal uptake of calcium and iron, each increase during pregnancy (Overduin et al., 2024). How macronutrient transporter expression and function change during pregnancy are less well described, however. Indeed, the reported changes in SI amino acid absorption during rat pregnancy are variable, with unchanged (Craft, 1970; Pénzes & Simon, 1968), increased, (Larralde & Fernandez Otero, 1968) and decreased (Dugas et al., 1970) absorption all described but with limited studies of each amino acid and no data for mice (Overduin et al., 2024). Transcript expression of the glucose‐galactose transporter sodium‐glucose co‐transporter 1 (*Slc5a1*, encoding SGLT1) and fructose transporter (S*lc2a5*, encoding GLUT5) increase 50% and 80%, respectively, at late pregnancy in the duodenum of rats relative to nonpregnant controls (Teerapornpuntakit et al., 2014). It is, however, unknown whether this occurs in other SI regions, at earlier pregnancy stages, or in other monogastric species. Consistent with the upregulation of carbohydrate transporters, glucose absorption per unit length of SI was 20%–40% higher in pregnant rats at gestational day (GD) 12–15 than in nonpregnant rats following intraluminal perfusion with 20–300 mM glucose (Larralde et al., 1966). Similarly, glucose absorption from ligated jejunal and ileal segments was 25% higher in anesthetized hamsters at late pregnancy compared to nonpregnant controls (Musacchia & Hartner, 1970). Few studies have directly measured glucose absorption in conscious pregnant animals, but portal vein glucose concentrations following a standardized meal were 20% higher in late‐pregnant compared to nonpregnant dogs (Moore et al., 2012), consistent with enhanced glucose absorption by the SI during pregnancy. Whether the abundance or function of fatty acid transporters CD36 and fatty acid binding protein 2 (FABP2), or the amino acid transporters SLC6A6 and SLC6A19, change during pregnancy has not been reported (Overduin et al., 2024).

Although the studies above provide evidence of anatomical and molecular adaptations in the SI at late pregnancy, the timing and regional changes, as well as the functional consequences, remain unclear. The current study, therefore, determined regional SI anatomy and expression of macronutrient transporters in non‐, early‐, mid‐, and late‐pregnant mice. Changes in carbohydrate transporter expression represented the largest effect of pregnancy and, therefore, we used optimized Ussing chamber methods to compare active glucose transport in mice at late pregnancy to that in nonpregnant mice in functional experiments.

## 2 MATERIALS AND METHODS

The original version of this manuscript, including methodology, was included in the PhD thesis awarded to Dr. T. Sebastian Overduin by the University of Adelaide in 2023 (Overduin, 2023).

### 2.1 Ethics

Experiments were approved by the Animal Ethics Committee of the South Australian Health and Medical Research Institute (SAHMRI, SAM395.19 & SAM‐21‐049). All studies were conducted in accordance with the Australian Code of Practice for the Care and Use of Animals for Scientific Purposes (National Health and Medical Research Council, 2013) and the ARRIVE guidelines (du Sert et al., 2020).

### 2.2 Study 1—Animals

Animal management was as previously described (Li et al., 2021). Briefly, 8‐week‐old C57BL/6J female mice, sourced from SAHMRI Bioresources, were individually housed in metabolic monitoring cages (Promethion Sable System, Las Vegas, NV) to acclimatize for 7 days under a 12‐h light cycle (0700–1900 h). Throughout the entire study, mice had access to standard laboratory chow (Teklad Global 18% protein Rodent Diet, Envigo, Cambridgeshire, UK; 18.6% crude protein; 6.2% fat; 44.2% carbohydrate) and water ad libitum. Mice were randomized to mating for pregnancy groups or not mated for age‐matched nonpregnant controls. Females for mating were housed in pairs with a C57BL/6J stud male at 1700 h. All females were checked every morning at 0700 h for a copulatory plug, which was indicative of successful mating and recorded as gestational day (GD) 0.5 of pregnancy. Following mating, plugged females were returned to metabolic cages and randomized to pregnancy‐stage groups. Final numbers of pregnant females at each pregnancy stage were early‐ (GD6.5, *n* = 10), mid‐ (GD12.5, *n* = 10), or late‐stage pregnancy (GD17.5, *n* = 11). Nonpregnant controls (*n* = 12) were transferred to metabolic cages on age‐matched days. Energy expenditure was determined by indirect calorimetry using both VO_2_ (oxygen consumption) and VCO_2_ (carbon dioxide production) measurements (Ladyman et al., 2018). Pregnant (GD 6.5, 12.5 or 17.5) and age‐matched nonpregnant females were anesthetized with isoflurane (5% in oxygen) and humanely killed via exsanguination with terminal collection of abdominal aorta blood.

### 2.3 Study 1—Small intestine histology

A laparotomy was performed, the SI was excised, and total weight was recorded. A single segment ~1 cm in length was resected from the middle of the duodenum 3–4 cm distally from the pyloric sphincter, the jejunum 10–11 cm distally from the pyloric sphincter, and the ileum 5–6 cm proximally from the ileocecal valve, respectively. Tissue segments were flushed with PBS, then fixed in 4% paraformaldehyde for 2 h, prior to overnight cryoprotection in 30% sucrose in 0.1 M phosphate buffer; tissues were then mounted in optimal cutting temperature (OCT) compound (ProSciTech, Thuringowa, Qld, Australia) and snap frozen in liquid nitrogen. OCT‐embedded intestinal segments were sectioned at 10 μm (Cryocut 1800; Leica Biosystems, Nussloch, Germany) and mounted on Superfrost Plus slides (Thermo Fisher, Waltham, MA, USA). Slides were stained with Hematoxylin and Eosin, then imaged at high resolution (Nanozoomer; Hamamatsu, Hamamatsu City, Shizuoka Prefecture, Japan). Villi height, crypt depth, and muscle thickness were measured using NDP Viewer 2.0 software (Hamamatsu). Up to 40 measurements of villi height, crypt depth, and smooth muscle thickness were averaged for each small intestinal region from each animal.

### 2.4 Study 1—Gene expression

Fresh SI mucosa was collected into an Eppendorf tube from a separate ~1 cm segment of each region by gently scraping from the serosa with a sterile scalpel blade. The tissue was then immediately snap‐frozen in liquid nitrogen and banked at −80°C until use. Mucosal mRNA from each SI region was extracted using a PuraLink RNA mini kit (Invitrogen, 12183018A) according to the manufacturer's instructions and mRNA yield and quality were determined using a NanoDrop (NanoDrop Technologies, Wilmington, DE, USA). Expression of carbohydrate (*Slc5a1*, *Slc2a2*, and *Slc2a5*), amino acid (*Slc15a1*, *Slc6a6*, and *Slc6a19*), and fatty acid (*Cd36* and *Fabp2*) transporter transcripts was determined for each SI region and pregnancy stage via quantitative real‐time qRT‐PCR utilizing Taqman and SYBR green according to the manufacturer's protocols (Table 1). Expression of *Fabp2* was calculated relative to the geometric mean transcript expression of reference genes β_2_ microglobulin (*B2m*) and hypoxanthine guanine phosphoribosyltransferase 1 (*Hprt1*). Expression of all other targets was calculated relative to the geometric mean transcript expression of reference genes β_2_ microglobulin (*B2m*), peptidylprolyl isomerase A (*Ppia*), and hypoxanthine guanine phosphoribosyltransferase 1 (*Hprt1*). NormFinder software was used to choose these reference genes with abundant SI expression that was stable across pregnancy (Li et al., 2018). The final NormFinder values for combined *B2m*, *Ppia*, and *Hprt1* were 0.005 in duodenum, 0.008 in jejunum, and 0.002 in ileum for Taqman, and 0.001 in duodenum–ileum and 0.002 in jejunum for SYBR. TaqMan qRT‐PCR was performed using a 7500 PCR thermocycler instrument (Applied Biosystems 7500 Real‐Time PCR System, Thermo Fisher Scientific) using EXPRESS One‐Step Superscript RTPCR kits (Invitrogen 11781‐200). SYBR green qRT‐PCR was performed using QuantiTect SYBR Green RT‐PCR kits (204245, Qiagen, Hilden, Germany) and QuantStudio 7 Flex Real‐Time PCR System (Thermo Fisher Scientific). Each assay was performed in triplicate and included no‐template and no‐RT controls. mRNA expression was quantified utilizing the delta CT method (Bookout & Mangelsdorf, 2003).

**TABLE 1:** Gene targets and assays used for qRT‐PCR study.

Gene name, *symbol* (also known as)	Function of encoded protein	Gene expression assay^a^	Protocol type
Solute carrier family 5 member 1, *Slc5a1* (Sodium‐glucose co‐transporter 1, *Sglt1*)	Primary SI apical glucose transporter	Mm00451203_M1	Taqman
Solute carrier family 2 member 2, *Slc2a2* (Glucose transporter 2, *Glut2*)	SI basolateral bi‐directional symporter for glucose/fructose	Mm00446229_M1	Taqman
Solute carrier family 2 member 5, *Slc2a5* (Glucose transporter 5, *Glut5*)	SI apical fructose transporter	Mm00600311_M1	Taqman
Solute carrier family 15 member 1, *Slc15a1* (Peptide transporter 1, *Pept1*)	Apical polypeptide transporter (≥ 3 amino acids)	Mm0451610_M1	Taqman
Solute carrier family 6 member 6, *Slc6a6*	Sodium‐chloride‐dependent taurine transporter	Mm00436909_M1	Taqman
Solute carrier family 6 member 19, *Slc6a6*	Sodium‐dependent neutral amino acid transporter	Mm01352157_M1	Taqman
Cd36 molecule, *Cd36*	SI apical fatty acid transporter	Mm00432403_M1	Taqman
Fatty acid binding protein 2, intestinal, *Fabp2*	Intracellular fatty acid transporter	Mm_Fabp2_1_SG	SYBR green
Beta‐2 microglobulin, *B2m*	Reference gene	Mm00437762_M1 Mm_B2m_2_SG	Taqman SYBR green
Hypoxanthine‐guanine phosphoribosyl transferase 1, *Hprt1*	Reference gene	Mm01545399_M1 Mm_Hprt_1_SG	Taqman SYBR green
Peptidylprolyl isomerase A, *Ppia*	Reference gene	Mm02342429_G1	Taqman

^a^ Gene expression assays used in Taqman protocols were purchased from ThermoFisher Scientific. Gene expression assays used with SYBR green protocols were purchased from Qiagen.

### 2.5 Study 2—Animals

Female 8–10‐week‐old C57BL/6J mice and proven‐fertile male C57BL/6J mice were sourced from SAHMRI Bioresources. All mice were acclimatized for a minimum of 7 days under a 12‐hour light cycle (0700–1900 h), weighed daily, and fed ad libitum as for Study 1. Following acclimatization, mice were randomized to mating to generate pregnant animals (*n* = 20) or not mated for age‐matched nonpregnant controls (*n* = 20). Females for mating were housed in pairs with a C57BL/6J stud male at 1700 h and checked for the presence of a copulatory plug at 0700 h each morning, which, when present, was recorded as GD0.5 of pregnancy. Female mice were pair‐housed in standard cages for the remainder of the study. Because active glucose transport differs by oestrous cycle stage in mice (Overduin et al., 2023), vaginal smears were assessed daily for at least a week prior to Ussing experiments, and mice in diestrus were used for nonpregnant studies (Caligioni, 2009). Data from a single nonpregnant mouse and a single pregnant mouse were excluded due to technical failure of the Ussing chamber platform, and three mated mice were excluded as they were either not pregnant or at the wrong day of pregnancy when humanely killed.

### 2.6 Study 2—Ex vivo active glucose transport

Mice at late pregnancy (GD17.5) and nonpregnant diestrus females were humanely killed via cervical dislocation. The abdominal cavity was opened along the midline, and the SI was exposed to ice‐cold Krebs‐Ringer bicarbonate buffer (KRB) fortified with 5 mM glucose. The full length of the SI was then removed, length measured, and the SI was divided into its constituent regions, with the transition from duodenum to jejunum marked by the ligament of Treitz (Mateer et al., 2016). The jejunum and ileum were separated based on gross morphological differences including a thinner muscle layer, paler appearance, and greater numbers of Peyer's patches in the ileum (Mateer et al., 2016). The length of each region was measured; then four segments ~1 cm long from each SI region (the 2 most proximal and 2 most distal segments) were collected from two SI regions per mouse. Individual mice were randomized to collection of each combination of sections (duodenum + jejunum, duodenum + ileum, or jejunum + ileum) providing eight combinations of tissue and conditions (region, distal/proximal segment, with/without phlorizin) across the eight Ussing chambers. Tissues from the proximal and distal halves of each collected region were mounted between two sliders with an exposed aperture of 0.1 cm^2^ and mounted into Ussing chambers (Overduin et al., 2023). Water‐jacketed chambers were filled with KRB buffer, continuously gassed with carbogen, and temperature maintained at 37°C throughout the experiment. Tissues from each region were randomized to equilibration and preincubation in the absence or presence of 0.3 mM phlorizin (#11576, Cayman Chemical Company) for 20 min, followed by exposure to luminal sided 45 mM D‐glucose (Overduin et al., 2023). Tissues were voltage‐clamped to zero potential difference, and short circuit current (*I*sc) monitored and recorded continuously (Acquire and Analyze 2.3, v2.3.4, Physiologic Instruments) throughout the experiment (Overduin et al., 2023). The absolute difference in short circuit current, expressed as Δ*I*sc (μA/cm^2^), was calculated as the difference between the maximum *I*sc reached during the 4 min following D‐glucose challenge and the average *I*sc during the 2 min prior to the D‐glucose challenge (Overduin et al., 2023). At the end of each experiment, tissue viability was assessed via a luminal sided 100 μM carbachol challenge. Only data from viable tissue that met quality control criteria (Overduin et al., 2023) was included in the analysis.

### 2.7 Statistics

SI weight, body weight, and litter size were compared between pregnancy stages by one‐way ANOVA. Effects of pregnancy stage (non, early, mid, and late), SI region (duodenum, jejunum, and ileum) and interactions on SI morphology and nutrient transporter gene expression were analyzed using mixed models, treating data from multiple regions from individual mice as repeated measures. Where interactions were present, effects of pregnancy stage were assessed separately within each region by one‐way ANOVA. Bonferroni post hoc tests were used to compare SI regions and pregnancy stages where a significant overall effect was identified. Active glucose transport data was log‐transformed for analysis and analyzed by mixed models for effects of pregnancy (non‐ or late‐pregnant), SI region (proximal duodenum, distal duodenum, proximal jejunum, distal jejunum, proximal ileum, distal ileum), phlorizin concentration (0, 0.3 mM) and all interactions. All statistical analyses were performed in SPSS version 28 (IBM Corporation, Armonk, New York, USA). Statistical significance was accepted when *p* ≤ 0.05, and all data are presented as mean ± standard deviation (SD).

## 3 RESULTS

The original version of this manuscript, including data, was included in the PhD thesis awarded to Dr. T. Sebastian Overduin by the University of Adelaide in 2023 (Overduin, 2023).

### 3.1 Study 1—Mouse and SI characteristics

Mice did not differ in initial body weight between groups, but by mid‐pregnancy were 24%–25% heavier than nonpregnant or early pregnant mice, while late‐pregnant mice were 57% heavier than nonpregnant mice (Table 2). Litter size was similar in all pregnant groups (Table 2). SI wet weight was similar in nonpregnant and early pregnant mice, but 20% heavier in mid‐ and late‐pregnant mice compared to nonpregnant controls (each *p <* 0.001, Table 2). Daily energy expenditure was 16%–18% higher in late‐pregnant than non‐ or early‐pregnant mice (Table 2).

**TABLE 2:** Study 1 mouse characteristics.

	Nonpregnant	Early pregnancy	Midpregnancy	Late pregnancy	Significance
No. of mice	11	10	10	11	
Gestational age	N/A	GD6.5	GD12.5	GD17.5	
Litter size	N/A	9.2 ± 0.6	8.6 ± 1.2	7.4 ± 2.7	0.074^d^
Initial body weight^a^ (g)	21.2 ± 0.7	20.7 ± 0.9	20.9 ± 0.9	20.9 ± 1.4	0.734
Final body weight^b^ (g)	22.1 ± 1.1^A^	22.4 ± 1.1^A^	27.7 ± 1.8^B^	34.8 ± 3.5^C^	**<0.001**
SI weight (g)	1.29 ± 0.15^A^	1.38 ± 0.08^A^	1.55 ± 0.08^B^	1.55 ± 0.10^B^	**<0.001**
Daily total energy expenditure^c^ (kcal)	9.5 ± 0.9^A^	9.3 ± 0.6^A^	10.2 ± 1.8^AB^	11.0 ± 1.4^B^	**0.010**

*Note*: Maternal data, litter size, and SI weight were analyzed by one‐way ANOVA, and Bonferroni comparison was used to compare specific pregnancy stages. Data are presented as mean ± SD. Significant differences are indicated in bold. Pregnancy stages that do not share a common superscript are different (^A,B,C^ *p <* 0.05).

Abbreviations: GD, gestational day; N/A, not applicable; NS, not significant; SI, small intestine.

^a^ Initial body weight was the average of body weight measures in metabolic cages during the last 48 h of acclimatization.

^b^ Final body weight was the average of body weight measures in metabolic cages during the 24 h before humane killing.

^c^ Daily total energy expenditure was the average obtained by indirect calorimetry in metabolic cages during the 48 h before humane killing.

^d^ Litter sizes were compared among pregnant groups only, and no pairs of groups differed in post‐hoc comparisons.

### 3.2 Study 1—SI morphology

**FIGURE 1:**
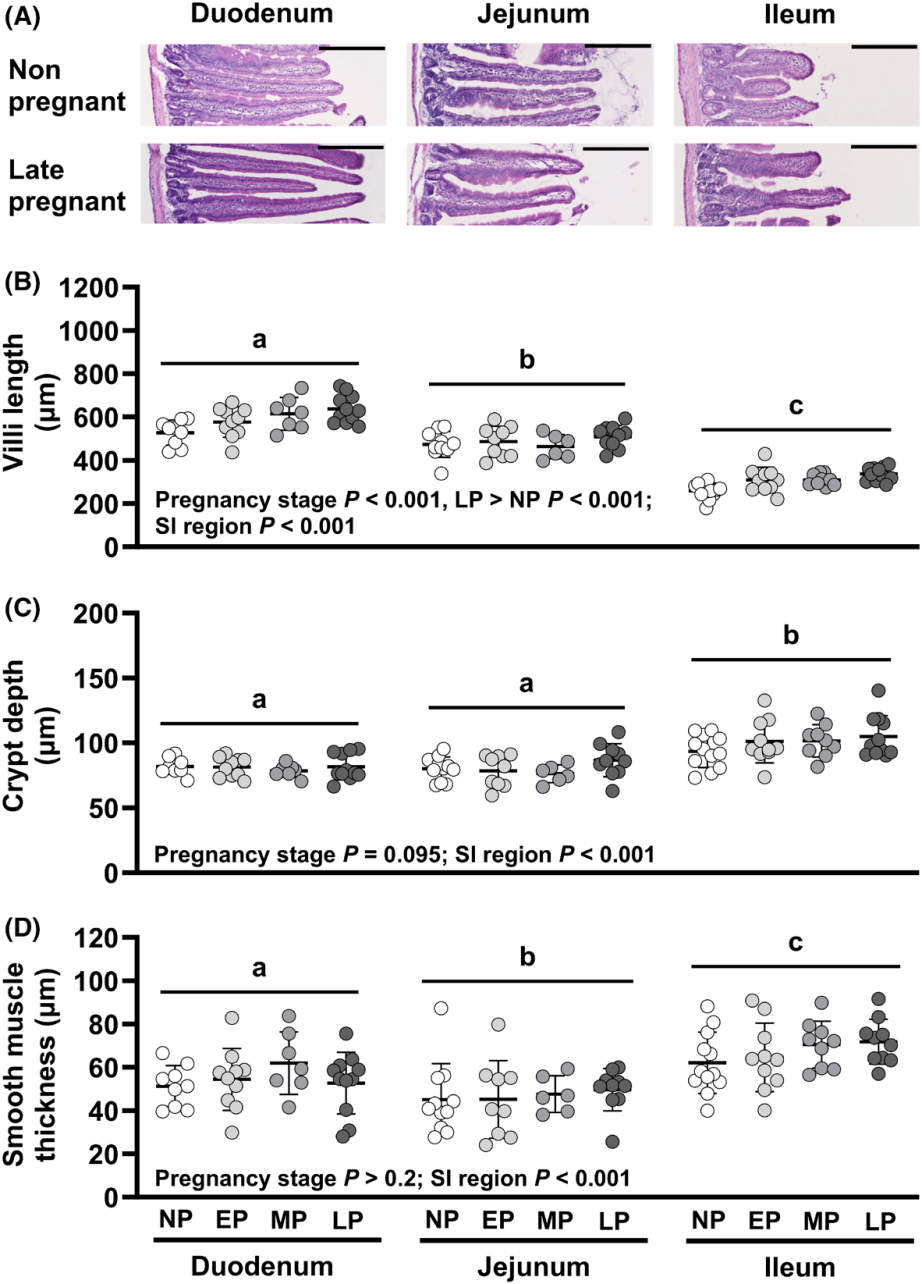
Small intestinal (SI) morphology. (A) Representative sections for each region from nonpregnant and late pregnant mice. Effects of pregnancy stage and SI region on (B) villi length, (C) crypt depth, and (D) smooth muscle thickness in C57BL/6J mice analyzed using a mixed model and treating data from the same mouse as repeated measures. Bars indicate mean ± SD, with data for individual animals shown by symbols. *N* = 6–12 mice per pregnancy stage. Overall differences (*p <* 0.05) between SI regions are indicated by letters (a, b, and c). *p* Values for each pair‐wise comparison are provided in Appendix S1. EP, early pregnant; LP, late pregnant; MP, mid pregnant; NP, nonpregnant.

The length of villi (Figure 1A) differed between pregnancy stages (*p <* 0.001) and between SI regions (*p <* 0.001), with similar effects of pregnancy stage in each region. Overall, villi were 18% longer in late‐pregnant mice (*p <* 0.001) but similar in early‐ (*p =* 0.158) and mid‐pregnant (*p =* 0.083) mice, compared to nonpregnant mice. Villi were longer in the duodenum than jejunum (21% difference, *p <* 0.001) or ileum (96% difference, *p <* 0.001) and 62% longer in jejunum than ileum (*p <* 0.001). Crypt depth (Figure 1B) also differed between SI regions (*p <* 0.001) but not between pregnancy stages (pregnancy stage *p =* 0.095; pregnancy stage*region *p >* 0.5). Crypt depth was similar in the duodenum and jejunum, but was 23% deeper in ileum than duodenum or jejunum (each *p <* 0.001). The thickness of the smooth muscle layer (Figure 1C) differed between regions (*p <* 0.001) but not between pregnancy stages (pregnancy stage *p >* 0.2; pregnancy stage*region *p >* 0.4). The smooth muscle layer was thinnest in the jejunum, intermediate in duodenum, and thickest in the ileum (*p <* 0.05 for each comparison).

### 3.3 Study 1—Nutrient transporter gene expression

#### 3.3.1 Carbohydrate transporters

**FIGURE 2:**
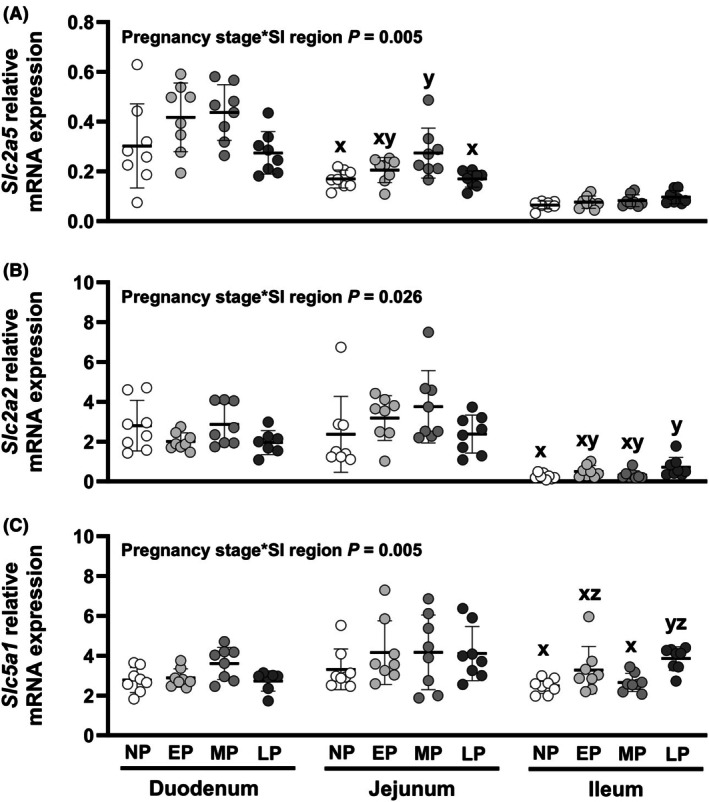
Relative transcript expression of carbohydrate transporters (A) *Slc2a5*, (B) *Slc2a2*, and (C) *Slc5a1* in the small intestine (SI). Effects of pregnancy stage and SI region were analyzed using a mixed model and treating data from the same mouse as repeated measures. Where interactions were present, effects of pregnancy stage were assessed separately within each SI region by one‐way ANOVA. Bars indicate mean ± SD, with data for individual animals shown by symbols. *N* = 7–8 mice per pregnancy stage. Differences (*p <* 0.05) between pregnancy stages within SI regions are indicated by letters (x, y, and z). *p* Values for each pair‐wise comparison are provided in Appendix S1. EP, early pregnant; LP, late pregnant; MP, mid pregnant; NP, nonpregnant.

Effects of pregnancy stage on relative expression of *Slc2a5* (GLUT5), *Slc2a2* (GLUT2), and *Slc5a1* (SGLT1) differed between SI regions (Figure 2A–C, pregnancy*region interactions each *p <* 0.05). *Slc2a5* expression (Figure 2A) differed by pregnancy stage in the duodenum (*p* = 0.041) and jejunum (*p* = 0.006) but not ileum (*p =* 0.069). Despite an overall effect of pregnancy stage, duodenal *Slc2a5* expression did not differ between pregnancy stages in pairwise comparisons. In the jejunum, *Slc2a5* expression was 1.7‐fold higher in mid‐pregnant compared to nonpregnant or late‐pregnant groups (Figure 2A, each *p =* 0.012). *Slc2a2* expression (Figure 2B) differed by pregnancy stage in the ileum (*p* = 0.029) but not duodenum (*p* = 0.107) or jejunum (*p* = 0.213). In the ileum, *Slc2a2* expression was 2.8‐fold higher in late‐pregnant compared to nonpregnant mice (Figure 2B, *p* = 0.039) but did not differ between other stages in pairwise comparisons. *Slc5a1* expression (Figure 2C) differed by pregnancy stage in the duodenum (*p* = 0.030) and ileum (*p* = 0.003) but not jejunum (*p* = 0.607). Despite an overall effect of pregnancy stage, duodenal *Slc5a1* expression did not differ between pregnancy stages in pairwise comparisons. In the ileum, *Slc5a1* expression (Figure 2C) was higher in late‐pregnant compared to nonpregnant (1.6‐fold, *p* = 0.004) or mid‐pregnant (1.5‐fold, *p* = 0.015) mice.

#### 3.3.2 Amino acid transporters

**FIGURE 3:**
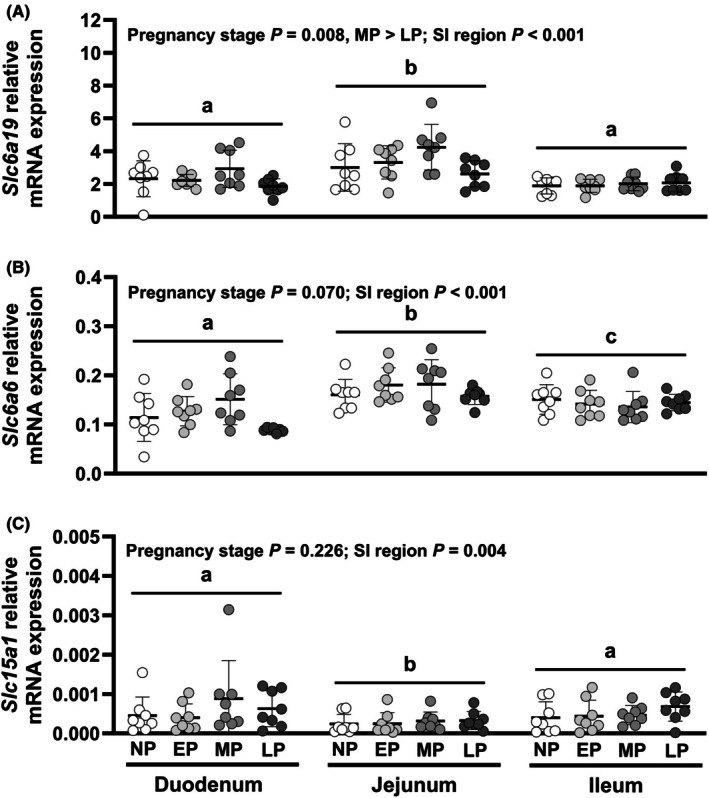
Relative transcript expression of amino acid transporters (A) *Slc6a19*, (B) *Slc6a6*, and (C) *Slc15a1* in the small intestine (SI). Effects of pregnancy stage and SI region were analyzed using a mixed model and treating data from the same mouse as repeated measures. Bars indicate mean ± SD, with data for individual animals shown by symbols. *N* = 7–8 mice per pregnancy stage. Differences (*p <* 0.05) between SI regions are indicated by letters (a, b, and c). *p* Values for each pair‐wise comparison are provided in Appendix S1. EP, early pregnant; LP, late pregnant; MP, mid pregnant; NP, nonpregnant.

Relative expression of the neutral amino acid transporter *Slc6a19* (Figure 3A) differed by SI region (*p <* 0.001) and pregnancy stage (*p =* 0.008), and effects of pregnancy stage did not differ between regions (interaction *p =* 0.113). S*lc6a19 e*xpression was 1.6‐fold higher in mid‐pregnant than late‐pregnant mice (*p =* 0.006), while expression in non‐ and early‐pregnant mice did not differ from other stages (Figure 3A). *Slc6a19* expression was 1.7‐ and 1.4‐fold higher in jejunum than duodenum or ileum, respectively (each *p <* 0.001). Relative expression of the taurine transporter *Slc6a6* (Figure 3B) differed by SI region (*p <* 0.001) but not by pregnancy stage (*p =* 0.070), and effects of pregnancy stage did not differ between regions (interaction *p =* 0.103). *Slc6a6 e*xpression was 1.4‐fold higher in jejunum than duodenum (*p <* 0.001). *Slc6a6 e*xpression in ileum was intermediate and different from both jejunum (*p =* 0.004) and duodenum (*p =* 0.030). Relative expression of peptide transporter 1 *Slc15a1* (Figure 3C), also known as *Pept1*, differed by SI region (*p =* 0.004) but not by pregnancy stage (*p =* 0.226). *Slc15a1* expression was 1.9‐fold lower in jejunum compared to duodenum (*p =* 0.030) or ileum (*p =* 0.024).

#### 3.3.3 Fatty acid transporters

**FIGURE 4:**
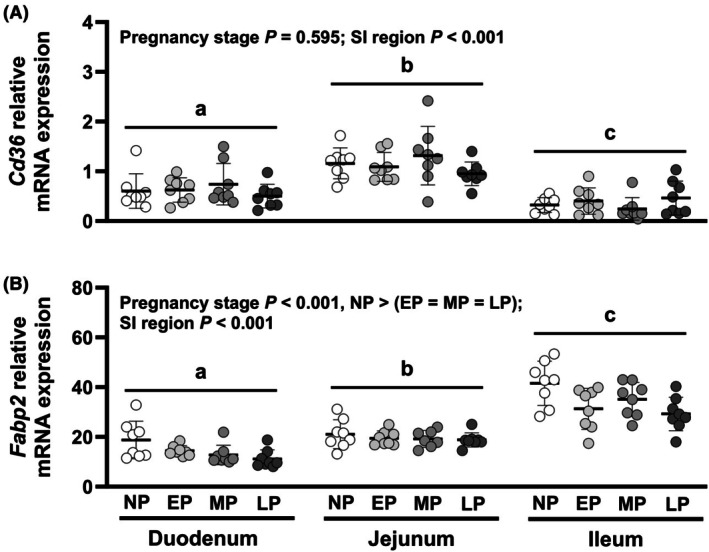
Relative transcript expression of fatty acid transporters (a) *Cd36* and (B) *Fabp2* in the small intestine (SI). Effects of pregnancy stage and SI region were analyzed using a mixed model and treating data from the same mouse as repeated measures. Bars indicate mean ± SD, with data for individual animals shown by symbols. *N* = 7–8 mice per pregnancy stage. Differences (*p <* 0.05) between SI regions are indicated by letters (a, b, and c). *p* Values for each pair‐wise comparison are provided in Appendix S1. EP, early pregnant; LP, late pregnant; MP, mid pregnant; NP, nonpregnant.

Relative expression of *Cd36* (Figure 4A) differed by SI region (*p <* 0.001) but not by pregnancy stage (*p =* 0.595), and effects of pregnancy stage did not differ between regions (interaction *p >* 0.1). *Cd36* expression was 3.1‐ and 1.8‐fold higher in jejunum than ileum or duodenum, respectively (each *p <* 0.001), and 1.7‐fold higher in the duodenum than ileum (*p =* 0.002). Relative expression of *Fabp2* (Figure 4B) differed by pregnancy stage and region (each *p <* 0.001), and effects of pregnancy stage did not differ between regions (interaction *p >* 0.1). *Fabp2* expression was 1.2‐ to 1.4‐fold higher in nonpregnant mice compared to pregnant mice at each stage of pregnancy (all *p <* 0.05) and did not differ between early‐, mid‐, or late‐pregnant groups. *Fabp2 e*xpression was 2.4‐fold higher in ileum than duodenum, 1.7‐fold higher in ileum than jejunum, and 1.4‐fold higher in jejunum than duodenum (each *p <* 0.001).

### 3.4 Study 2—Mouse characteristics and SI gross morphology

Mice did not differ in initial body weight between groups, while final weights of late‐pregnant mice were >50% heavier than nonpregnant mice (*p <* 0.001, Table 3). The total SI, duodenum, and ileum were each 4%–5% longer in late‐pregnant than nonpregnant mice (each *p <* 0.05), while jejunum lengths did not differ between these groups (Table 3).

**TABLE 3:** Study 2 mouse characteristics.

	Nonpregnant	Late pregnancy	Significance
No. of mice	19	16	
Gestational age	N/A	GD17.5	N/A
Litter size	0	7.4 ± 1.9	N/A
Initial body weight^a^ (g)	20.1 ± 1.9	20.0 ± 1.8	0.806
Final body weight^b^ (g)	21.1 ± 1.6	32.0 ± 5.1	**<0.001**
SI lengths (cm)			
Total	31.9 ± 1.7	33.3 ± 1.8	**0.027**
Duodenum	7.7 ± 0.5	8.1 ± 0.5	**0.028**
Jejunum	9.5 ± 0.6	9.9 ± 0.8	0.123
Ileum	14.7 ± 0.9	15.3 ± 0.7	**0.035**

*Note*: Data were compared between nonpregnant and pregnant groups by 1‐way ANOVA and are presented as mean ± SD. Significant differences are indicated in bold.

Abbreviations: GD, gestational day; N/A, not applicable; SI, small intestine.

^a^ Initial body weight was the body weight recorded at the start of the 7‐day acclimatization period.

^b^ Final body weight was the body weight recorded on the morning of experiments directly prior to humane killing.

### 3.5 Study 2—Active glucose transport

**FIGURE 5:**
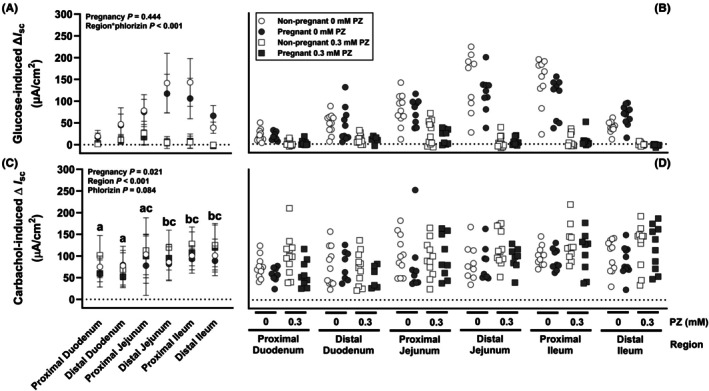
Active glucose transport in the small intestine (SI) of late‐pregnant and diestrus mice, with and without inhibition of SGLT1‐mediated active glucose transport using phlorizin (PZ). Effects of pregnancy, SI region, and PZ were analyzed using a mixed model and treating data from the same mouse as repeated measures. Data are presented as group mean ± SD (A and C) and individual values (B and D). *N* = 16–19 mice per pregnancy stage. Differences (*p <* 0.05) between SI regions are indicated by letters (a, b, and c). *p* Values for each pair‐wise comparison are provided in Appendix S1.

Active glucose transport (Figure 5A,B) did not differ between nonpregnant and late‐pregnant mice (*p >* 0.4), while the effects of the SGLT1 inhibitor phlorizin and SI region were interdependent (interaction: *p <* 0.001). In tissues incubated without phlorizin, glucose‐induced Δ*I*_sc_ differed between SI regions (*p <* 0.001) and was 2.7‐ to 7.0‐fold higher in all other regions compared to the proximal duodenum (all *p <* 0.01). Glucose‐induced Δ*I*_sc_ in the absence of phlorizin was ~2.5‐fold higher in distal jejunum and proximal ileum than distal duodenum and distal ileum (all *p <* 0.002), while responses in proximal jejunum were intermediate and not different from any other region except proximal duodenum. Phlorizin inhibited glucose‐induced Δ*I*_sc_ by between 70% and 99% in all regions (each *p <* 0.01). In the presence of phlorizin, glucose responses differed between SI regions (*p <* 0.001), being >5‐fold lower in distal ileum than in all other regions except proximal duodenum (all *p* ≤ 0.031). Carbachol‐induced Δ*I*_sc_ (Figure 5C,D) was ~17% lower in late‐pregnant than in nonpregnant mice (*p <* 0.05) and differed between regions (*p <* 0.001) but was unaffected by phlorizin (*p =* 0.084), with no interactions between factors. Carbachol‐induced Δ*I*_sc_ was ~1.4‐fold greater in distal jejunum, proximal ileum, and distal ileum than in either duodenal region (each *p <* 0.05) and intermediate in proximal jejunum.

## 4 DISCUSSION

The findings of this study provide new evidence that the SI undergoes structural (length and weight), micro‐structural (villi morphology) and transcriptional changes in nutrient transporter expression that would be expected to facilitate increased nutrient uptake in murine pregnancy. Food intake, weight, and energy expenditure all increased by mid‐late pregnancy in these mice (Li et al., 2021), supporting maternal, fetal, and placental weight gains. Despite these SI changes, active glucose transport per unit area was similar within a given SI region irrespective of pregnancy stage, in contrast to reports of increased uptake across the whole SI or SI segments in mid‐ to late‐pregnant rats and hamsters (Overduin et al., 2024). Whether other glucose‐transport mechanisms change during pregnancy, or effects of pregnancy on glucose transport depend on nutritional status, requires further investigation.

The moderate SI elongation in murine pregnancy was consistent with existing reports in rodents (Overduin et al., 2024) and adds new knowledge that increased overall SI length is due to increases in duodenum and ileum length alone. This is consistent with evidence of increased length of the combined jejunum and ileum in late‐pregnant rats (Burdett & Reek, 1979) and increased duodenum length in late‐pregnant shrews (Jaroszewska & Wilczyńska, 2006), although our study is the first to report data for multiple SI regions. We also provide new data on SI microstructure during mouse pregnancy, addressing gaps and inconsistencies in the data underlying descriptions of increased overall SI weight and length and of villi length during pregnancy (Astbury et al., 2015). Indeed, adaptations to SI microstructure appear to be variable between studies, SI regions, and pregnancy stages (Overduin et al., 2024). In contrast to our finding that villi were 18% longer in late pregnancy compared to nonpregnant animals, regardless of SI region, unchanged SI villi lengths were reported in pregnant compared to nonpregnant common shrews (Jaroszewska & Wilczyńska, 2006). Others have reported inconsistent and stage‐specific region‐specific changes in villi length during rodent pregnancy, which were either increased (Cripps & Williams, 1975) or unchanged (Sabet Sarvestani et al., 2015) in the rat duodenum, increased in rat and mouse jejunum (Sabet Sarvestani et al., 2015; Sensoy & Oznurlu, 2019), and unchanged (Cripps & Williams, 1975) or decreased (Sabet Sarvestani et al., 2015; Sensoy & Oznurlu, 2019) in the ileum. Likewise, variable changes in crypt depth and smooth muscle thickness have been reported between studies, on a limited evidence base (Overduin et al., 2024), and neither parameter changed in the present study. The reasons for these differences between studies are unclear. It is possible that hypertrophy of the SI and nutrient uptake itself vary depending on the extent of maternal adaptation needed to support pregnancy. Currently, however, there is little morphological data available during pregnancy for monogastric species in which fetal weight is proportionately less relative to maternal weight (Overduin et al., 2024). In addition to studies in larger animal species, investigating SI adaptations in pregnant dams with a wide range of litter sizes and hence increases in demand for nutrient supply may be useful in investigating the relationship between conceptus demand and SI adaptation. Together, the elongation of the entire SI and its villi that we observed was expected to expand SI surface area, although it was not possible to assess this directly in the current study. However, in a single report on internal surface areas of SI regions in shrews, calculated from SI size, villi length, and density, surface areas did not change during pregnancy, while duodenal internal surface area increased during lactation (Jaroszewska & Wilczyńska, 2006).

To the best of our knowledge, this study is the first to map regional transcript expression of SI macronutrient transporters during pregnancy. We found expression of glucose transporters *Slc5a1* (*SGLT* *1*) and *Slc2a2* (*GLUT2*) was augmented in only the ileum in mice at late pregnancy, with no changes observed earlier in pregnancy or in other SI regions. This lack of change in duodenal *Slc5a1* expression contrasts with the only other report of pregnancy‐related SI carbohydrate transporter expression in rodents, which only assessed the duodenum and reported a 1.5‐fold higher *Slc5a1* expression in duodenal mucosa of late‐ compared to nonpregnant rats (Teerapornpuntakit et al., 2014). The reasons for these differential responses are unclear, since animals were fed a standard chow and were unfasted in both studies, although since the timing of tissue collection was not stated in the former study (Teerapornpuntakit et al., 2014), it is possible that pregnancy‐related changes differ with circadian cycle. Indeed, we have reported that increases in food intake and meal size during late pregnancy occurred mostly in the light phase in mice (Li et al., 2021). Similar jejunal *Slc5a1* expression in pregnant and nonpregnant mice in the present study may indicate that jejunal capacity for carbohydrate uptake is near maximal in nonpregnant mice and cannot further upregulate. In nonpregnant rodents and humans, the final stages of carbohydrate digestion to produce simple sugars occur primarily in the duodenum, while the majority of glucose uptake occurs in the upper two‐thirds of the jejunum (Karasov, 2017; Volk & Lacy, 2017). Together with greater food intake during pregnancy, a ceiling on jejunal glucose uptake could increase ileal exposure to glucose. Increased exposure may then drive the 1.5‐fold and 1.8‐fold increases in ileal *Slc5a1* and *Slc2a2* expression, respectively, increasing ileal capacity for carbohydrate absorption relative to the nonpregnant state. Although expression of the basolateral glucose‐fructose transporter *Slc2a2* remained lower in the ileum than in the duodenum or jejunum during pregnancy, transcript expression of the apical glucose transporter *Slc5a1* was similar across SI regions. Upregulation of these transporters during pregnancy is consistent with the hypothesis that the ileum acts as a functional reserve for nutrient absorption capacity (Burdett & Reek, 1979). Carbohydrate absorption over a greater proportion of the SI could also extend the duration of carbohydrate absorption to sustain circulating blood glucose after each meal. This, in turn, could support placental glucose uptake and delivery to the fetus, which occurs down a concentration gradient (Dilworth & Sibley, 2013). In addition, increased ileal glucose may activate the ileal brake, triggering release of glucagon‐like peptide‐1 (GLP‐1), which acts to slow upper SI motility (Holst, 2007). Consistent with this hypothesis, fed concentrations of circulating GLP‐1 were ~4‐fold higher in mid‐ than nonpregnant rats, associated with gut hypertrophy but not satiety (Johnson et al., 2019). Reduced satiety actions of GLP‐1 may reflect the downregulation of central and peripheral satiety pathways during pregnancy, facilitating 20%–30% increases in food intake by late pregnancy in rodents (Clarke et al., 2021). Independent of satiety‐related actions, increased GLP‐1 secretion due to ileal brake activation may extend gastrointestinal transit time in late pregnancy, which is consistently increased by 20%–80% in women and rodents (Overduin et al., 2024).

In the present study, expression of amino acid transporters *Slc6a19*, *Slc6a6*, and *Slc15a1* transcripts was similar across pregnancy stages and SI regions. Impacts of pregnancy on SI amino acid uptake are not well characterized, although glycine absorption was 20% higher in late‐ compared to nonpregnant mice (Larralde & Fernandez Otero, 1968). Glycine uptake is mediated by proton‐coupled amino acid transporter 1 (PAT1), encoded by *Slc36a1* (Kiela & Ghishan, 2016) so it is possible that expression of this transporter may change at the transcript and/or protein level during pregnancy, which was not measured in the present study.

This study provides the first reported data on the expression of fatty acid transporters in the SI during pregnancy (Overduin et al., 2024). The expression of apical fatty acid transporter *Cd36* transcripts did not differ between pregnancy stages and was higher in the jejunum than in the duodenum or ileum. This contrasts with reports of similar CD36 protein abundance in the duodenum and jejunum of adult male C57BL/6 mice fed a normal‐fat (control) diet, while high‐fat feeding upregulated jejunal but not duodenal expression (Lynes et al., 2011). In contrast to the lack of effect of pregnancy on *Cd36*, we observed declining expression of the intracellular transporter *Fabp2* in the duodenum and ileum as pregnancy progressed. FABP2 traffics fatty acids to the endoplasmic reticulum for export into the circulation (Ko et al., 2020) and decreased FABP2 expression would likely reduce fatty acid supply from the SI, although uptake of fatty acids by the SI has not been reported in pregnancy. It is possible that fatty acids are diverted for use as energy sources within epithelial cells during pregnancy. This may be required to support SI growth, which is particularly marked in late pregnancy of rodents (Overduin et al., 2024). Switching the SI energy source from glucose to fatty acids would also protect glucose supply to the circulation to support conceptus development. In contrast to pregnancy‐related down‐regulation in the duodenum and ileum, *Fabp2* expression was unchanged in the jejunum during pregnancy, in parallel with the region‐specific pattern of adaptations to carbohydrate transporter expression.

Region‐specific gains in SI length, villi elongation, and expression of glucose transporters led us to expect augmented SI glucose transport in mice at late pregnancy, which we therefore measured by Ussing chamber methodology (Overduin et al., 2023). This method allows measurement of region‐specific active glucose uptake and investigation of specific transporter activity through use of inhibitors (Overduin et al., 2023). Importantly, ex vivo studies of SI glucose transport avoid the limitation of oral absorption studies in pregnancy, where changes in circulating nutrient abundance after intake will be confounded by placental nutrient uptake (Dilworth & Sibley, 2013). In our study, however, the higher ileal expression of *Slc5a1* and *Slc2a2* transcription during pregnancy did not equate to functional gains in active glucose transport, such that ex vivo active glucose transport across isolated SI segments was similar in late‐ and non‐pregnant mice. The absence of enhanced glucose transport in pregnancy in our study contrasts with studies using SI perfusion or everted sac methodology, in which glucose transfer across the epithelium was ~20%–30% higher in late‐pregnant compared to nonpregnant rodents (Larralde et al., 1966; Musacchia & Hartner, 1970). Increased SI size during pregnancy may also have contributed to increased glucose uptake in perfusion studies. It is also possible that differences in impacts of pregnancy on glucose uptake between studies may reflect our measure of active SGLT1‐mediated glucose transport, rather than overall glucose transfer. Increases in passive glucose transport and/or reduced SI tissue metabolism of glucose may occur during pregnancy, which would require evaluation using different methodologies. It is also possible that GLUT2 may play a greater role in glucose transport during pregnancy, since ileal expression was higher in late pregnancy, and GLUT2‐mediated glucose transport does not generate current, which is the outcome detected by the Ussing chamber methodology. There is some, albeit controversial, evidence for translocation of GLUT2 from the basal to apical membrane in extreme physiological states, including a cohort study of morbidly obese women (Ait‐Omar et al., 2011). Whether this occurs in healthy pregnancies has not been assessed. Finally, differences between studies may also reflect different nutritional status of the rodents, which were fasted for 4–24 h before tissue collection or perfusion in past studies (Amano, 1967; Larralde et al., 1966; Musacchia & Hartner, 1970), but had unrestricted access to food in the present study. *Slc5a1* expression is upregulated by luminal glucose (Koepsell, 2020), and therefore transcription may already be upregulated in nonpregnant as well as pregnant animals in the present study. It is possible that differential SGLT1 activity between pregnant and nonpregnant animals may be evident on fasting and may contribute to the higher glucose transport reported during pregnancy (Amano, 1967; Larralde et al., 1966; Musacchia & Hartner, 1970). Although not measured in the present study, slower gastrointestinal transit during pregnancy (Overduin et al., 2024) may also increase the opportunity for glucose uptake in vivo. Thus, the potential for changes in glucose transport during pregnancy requires further investigation and is likely to vary with nutritional state. While intestinal nutrient delivery is a key factor, we recognize that other mechanisms contribute to increased glucose availability in support of fetal and maternal requirements during pregnancy. Notably, in addition to decreased insulin sensitivity of maternal peripheral tissues in the second half of pregnancy (Lain & Catalano, 2007; Leturque et al., 1980), circulating glucose concentrations are maintained by elevated hepatic endogenous glucose production. This is 30% and 60% higher in late‐pregnant compared to nonpregnant women (Butte, 2000) and rats (Einstein et al., 2008), respectively, compared to their nonpregnant counterparts.

In conclusion, our study has provided fundamental insight into the SI region and pregnancy‐stage specific adaptations in anatomy and nutrient transporter transcription expression in mice. Increases in SI weight by mid‐pregnancy likely reflect elongation of the duodenum and ileum. Elongation of villi by late pregnancy occurred independent of the SI region and would be expected to increase the surface area for nutrient absorption. The mechanisms driving SI hypertrophy and other adaptations during pregnancy require additional investigation. Circulating hormone concentrations change markedly during pregnancy, including increases in progesterone, estrogens, leptin, placental lactogens, prolactin, and/or growth hormone, and the intestinal mucosa expresses receptors for many of these hormones, several of which are implicated in peripheral and central mechanisms allowing increases in food intake during pregnancy (Clarke et al., 2021). Upregulation of carbohydrate transporter expression was seen primarily in the ileum, where it may act in concert with gains in passive absorption (increased surface area) to support fetal development and the deposition of maternal reserves. Unexpectedly, given the anatomical and molecular changes, active SGLT1‐mediated glucose transport per unit area was not greater in late‐pregnant than age‐matched nonpregnant mice. Increased nutrient demand during pregnancy may therefore be met largely through SI expansion, although contributions of other nutrient transport mechanisms require evaluation. Precise knowledge of the adaptations will inform future research that optimizes nutritional and dietary support during human pregnancy, improves pregnancy outcomes, and safeguards fetal development and the future health of the mother and newborn.

## AUTHOR CONTRIBUTIONS

TSO, GSC, RLY, KLG, and AJP: Conceived and designed research. TSO, GSC, HL, KLG, and AJP: Performed experiments and analyzed data. TSO, GSC, HL, RLY, KLG, and AJP: Interpreted results of experiments. TSO and KLG: Prepared figures. TSO, KLG, and AJP: Drafted manuscript. GSC, RLY, KLG, and AJP: Edited and revised manuscript. All authors approved the final version of the manuscript.

## FUNDING INFORMATION

While conducting this work, G. S. Clarke held the Robinson Honours scholarship of the Robinson Research Institute, University of Adelaide, and subsequently an Australian Government Research Training Program (RTP) Stipend Scholarship. T. S. Overduin was supported by a Faculty of Health and Medical Sciences Scholarship.

## CONFLICT OF INTEREST STATEMENT

The authors have no conflict of interest to declare.

## Supporting information


Appendix S1.

## Data Availability

Data are available from the corresponding authors on reasonable request.

## References

[phy270493-bib-0001] Ait‐Omar, A. , Monteiro‐Sepulveda, M. , Poitou, C. , Le Gall, M. , Cotillard, A. , Gilet, J. , Garbin, K. , Houllier, A. , Château, D. , Lacombe, A. , Veyrie, N. , Hugol, D. , Tordjman, J. , Magnan, C. , Serradas, P. , Clément, K. , Leturque, A. , & Brot‐Laroche, E. (2011). GLUT2 accumulation in enterocyte apical and intracellular membranes: A study in morbidly obese human subjects and ob/ob and high fat‐fed mice. Diabetes, 60, 2598–2607. 21852673 10.2337/db10-1740 PMC3178286

[phy270493-bib-0002] Amano, Y. (1967). Changes in the levels of blood glucose during pregnancy in the rat. Japanese Journal of Pharmacology, 17, 105–114. 5299964 10.1254/jjp.17.105

[phy270493-bib-0003] Astbury, S. , Mostyn, A. , Symonds, M. E. , & Bell, R. C. (2015). Nutrient availability, the microbiome, and intestinal transport during pregnancy. Applied Physiology, Nutrition, and Metabolism, 40, 1100–1106. 10.1139/apnm-2015-0117 26499849

[phy270493-bib-0004] Bookout, A. L. , & Mangelsdorf, D. J. (2003). Quantitative real‐time PCR protocol for analysis of nuclear receptor signaling pathways. Nuclear Receptor Signaling, 1, e012. 16604184 10.1621/nrs.01012 PMC1402222

[phy270493-bib-0005] Burdett, K. , & Reek, C. (1979). Adaptation of the small intestine during pregnancy and lactation in the rat. The Biochemical Journal, 184, 245–251. 534527 10.1042/bj1840245 PMC1161758

[phy270493-bib-0006] Butte, N. F. (2000). Carbohydrate and lipid metabolism in pregnancy: Normal compared with gestational diabetes mellitus. The American Journal of Clinical Nutrition, 71, 1256s–1261s. 10799399 10.1093/ajcn/71.5.1256s

[phy270493-bib-0007] Caligioni, C. S. (2009). Assessing reproductive status/stages in mice. Current Protocols in Neuroscience, 48, A‐41. 10.1002/0471142301.nsa04is48 PMC2755182 19575469

[phy270493-bib-0008] Clapp, J. F., 3rd , Stepanchak, W. , Tomaselli, J. , Kortan, M. , & Faneslow, S. (2000). Portal vein blood flow‐effects of pregnancy, gravity, and exercise. American Journal of Obstetrics and Gynecology, 183, 167–172. 10920326 10.1067/mob.2000.105902

[phy270493-bib-0009] Clarke, G. S. , Gatford, K. L. , Young, R. L. , Grattan, D. R. , Ladyman, S. R. , & Page, A. J. (2021). Maternal adaptations to food intake across pregnancy: Central and peripheral mechanisms. Obesity, 29, 1813–1824. 34623766 10.1002/oby.23224

[phy270493-bib-0010] Craft, I. L. (1970). The influence of pregnancy and lactation on the morphology and absorptive capacity of the rat small intestine. Clinical Science, 38, 287–295. 5442552 10.1042/cs0380287

[phy270493-bib-0011] Cripps, A. W. , & Williams, V. J. (1975). The effect of pregnancy and lactation on food intake, gastrointestinal anatomy and the absorptive capacity of the small intestine in the albino rat. The British Journal of Nutrition, 33, 17–32. 1115751 10.1079/bjn19750005

[phy270493-bib-0012] Dilworth, M. R. , & Sibley, C. P. (2013). Review: Transport across the placenta of mice and women. Placenta, 34, S34–S39. 23153501 10.1016/j.placenta.2012.10.011

[phy270493-bib-0013] du Sert, P. , Hurst, V. , Ahluwalia, A. , Alam, S. , Avey, M. T. , Baker, M. , Browne, W. J. , Clark, A. , Cuthill, I. C. , Dirnagl, U. , Emerson, M. , Garner, P. , Holgate, S. T. , Howells, D. W. , Karp, N. A. , Lazic, S. E. , Lidster, K. , MacCallum, C. J. , Macleod, M. , … Wurbel, H. (2020). The ARRIVE guidelines 2.0: Updated guidelines for reporting animal research. PLoS Biology, 18, e3000410. 32663219 10.1371/journal.pbio.3000410 PMC7360023

[phy270493-bib-0014] Dugas, M. C. , Hazelwood, R. L. , & Lawrence, A. L. (1970). Influence of pregnancy and‐or exercise on intestinal transport of amino acids in rats. Proceedings of the Society for Experimental Biology and Medicine, 135, 127–131. 5475609 10.3181/00379727-135-35003

[phy270493-bib-0015] Einstein, F. H. , Fishman, S. , Muzumdar, R. H. , Yang, X. M. , Atzmon, G. , & Barzilai, N. (2008). Accretion of visceral fat and hepatic insulin resistance in pregnant rats. American Journal of Physiology. Endocrinology and Metabolism, 294, E451–E455. 18073320 10.1152/ajpendo.00570.2007

[phy270493-bib-0016] Holst, J. J. (2007). The physiology of glucagon‐like peptide 1. Physiological Reviews, 87, 1409–1439. 17928588 10.1152/physrev.00034.2006

[phy270493-bib-0017] Jaroszewska, M. , & Wilczyńska, B. (2006). Dimensions of surface area of alimentary canal of pregnant and lactating common female shrews. Journal of Mammalogy, 87, 589–597.

[phy270493-bib-0018] Johnson, M. L. , Saffrey, M. J. , & Taylor, V. J. (2019). Gastrointestinal capacity, gut hormones and appetite change during rat pregnancy and lactation. Reproduction, 157, 431–443. 30790767 10.1530/REP-18-0414

[phy270493-bib-0019] Karasov, W. H. (2017). Integrative physiology of transcellular and paracellular intestinal absorption. The Journal of Experimental Biology, 220, 2495–2501. 28724701 10.1242/jeb.144048

[phy270493-bib-0020] Kiela, P. R. , & Ghishan, F. K. (2016). Physiology of intestinal absorption and secretion. Best Practice & Research. Clinical Gastroenterology, 30, 145–159. 27086882 10.1016/j.bpg.2016.02.007 PMC4956471

[phy270493-bib-0021] Ko, C. W. , Qu, J. , Black, D. D. , & Tso, P. (2020). Regulation of intestinal lipid metabolism: Current concepts and relevance to disease. Nature Reviews. Gastroenterology & Hepatology, 17, 169–183. 32015520 10.1038/s41575-019-0250-7

[phy270493-bib-0022] Koepsell, H. (2020). Glucose transporters in the small intestine in health and disease. Pflügers Archiv, 472, 1207–1248. 32829466 10.1007/s00424-020-02439-5 PMC7462918

[phy270493-bib-0023] Ladyman, S. R. , Carter, K. M. , & Grattan, D. R. (2018). Energy homeostasis and running wheel activity during pregnancy in the mouse. Physiology & Behavior, 194, 83–94. 29738792 10.1016/j.physbeh.2018.05.002

[phy270493-bib-0024] Lain, K. Y. , & Catalano, P. M. (2007). Metabolic changes in pregnancy. Clinical Obstetrics and Gynecology, 50, 938–948. 17982337 10.1097/GRF.0b013e31815a5494

[phy270493-bib-0025] Larralde, J. , & Fernandez Otero, P. (1968). Effect of pregnancy on in vitro intestinal absorption of glucose and glycine. Revista Española de Fisiología, 24, 49–50. 5738201

[phy270493-bib-0026] Larralde, J. , Fernandez‐Otero, P. , & Gonzalez, M. (1966). Increased active transport of glucose through the intestine during pregnancy. Nature, 209, 1356–1357. 10.1038/2091356b0 5956056

[phy270493-bib-0027] Leturque, A. , Ferré, P. , Satabin, P. , Kervran, A. , & Girard, J. (1980). In vivo insulin resistance during pregnancy in the rat. Diabetologia, 19, 521–528. 7007133 10.1007/BF00253179

[phy270493-bib-0028] Li, H. , Clarke, G. S. , Christie, S. , Ladyman, S. R. , Kentish, S. J. , Young, R. L. , Gatford, K. L. , & Page, A. J. (2021). Pregnancy‐related plasticity of gastric vagal afferent signals in mice. American Journal of Physiology. Gastrointestinal and Liver Physiology, 320, G183–G192. 33206550 10.1152/ajpgi.00357.2020

[phy270493-bib-0029] Li, H. , Kentish, S. J. , Wittert, G. A. , & Page, A. J. (2018). Apelin modulates murine gastric vagal afferent mechanosensitivity. Physiology & Behavior, 194, 466–473. 29964068 10.1016/j.physbeh.2018.06.039

[phy270493-bib-0030] López‐Luna, P. , Maier, I. , & Herrera, E. (1991). Carcass and tissue fat content in the pregnant rat. Biology of the Neonate, 60, 29–38. 1912096 10.1159/000243385

[phy270493-bib-0031] Lynes, M. , Narisawa, S. , Millán, J. L. , & Widmaier, E. P. (2011). Interactions between CD36 and global intestinal alkaline phosphatase in mouse small intestine and effects of high‐fat diet. American Journal of Physiology. Regulatory, Integrative and Comparative Physiology, 301, R1738–R1747. 21900644 10.1152/ajpregu.00235.2011 PMC3233846

[phy270493-bib-0032] Mateer, S. W. , Cardona, J. , Marks, E. , Goggin, B. J. , Hua, S. , & Keely, S. (2016). Ex vivo intestinal sacs to assess mucosal permeability in models of gastrointestinal disease. Journal of Visualized Experiments, e53250. 26891144 10.3791/53250 PMC4781746

[phy270493-bib-0033] Moore, M. C. , Smith, M. S. , & Connolly, C. C. (2012). Pregnancy augments hepatic glucose storage in response to a mixed meal. British Journal of Nutrition, 107, 493–503. 21831337 10.1017/S0007114511003187 PMC3647386

[phy270493-bib-0034] Musacchia, X. J. , & Hartner, A. M. (1970). Intestinal absorption of glucose, and blood glucose and hematocrit in pregnant and nonpregnant hamsters. Proceedings of the Society for Experimental Biology and Medicine, 135, 307–310. 5529595 10.3181/00379727-135-35041

[phy270493-bib-0035] National Health and Medical Research Council . (2013). Australian code for the care and use of animals for scientific purposes (8th ed., p. 82). Australian Government Publishing Service.

[phy270493-bib-0036] Overduin, T. S. (2023). Adaptations of small intestinal nutrient absorption during pregnancy in mice. PhD thesis, School of Biomedicine, University of Adelaide. https://hdl.handle.net/2440/140564

[phy270493-bib-0037] Overduin, T. S. , Page, A. J. , Young, R. L. , & Gatford, K. L. (2024). Adaptations in gastrointestinal nutrient absorption and its determinants during pregnancy in Monogastric mammals: A scoping review. Nutrition Reviews, 83, e1172–e1196. 10.1093/nutrit/nuae064 38926118

[phy270493-bib-0038] Overduin, T. S. , Wardill, H. R. , Young, R. L. , Page, A. J. , & Gatford, K. L. (2023). Active glucose transport varies by small intestinal region and oestrous cycle stage in mice. Experimental Physiology, 108, 865–873. 10.1113/EP091040 37022128 PMC10988461

[phy270493-bib-0039] Pénzes, L. , & Simon, G. (1968). Intestinal absorption and turnover of DL‐methionine during reproduction in the rat. The Japanese Journal of Physiology, 18, 288–296. 5303533 10.2170/jjphysiol.18.288

[phy270493-bib-0040] Pere, M. C. , Baudelin, A. , Briggs, K. , & Gilbert, M. (1992). Hepatic metabolism during fasting‐refeeding transition in conscious pregnant rabbits. The American Journal of Physiology, 262, E899–E905. 1616023 10.1152/ajpendo.1992.262.6.E899

[phy270493-bib-0041] Sabet Sarvestani, F. , Rahmanifar, F. , & Tamadon, A. (2015). Histomorphometric changes of small intestine in pregnant rat. Veterinary Research Forum, 6, 69–73. 25992254 PMC4405688

[phy270493-bib-0042] Sensoy, E. , & Oznurlu, Y. (2019). Determination of the changes on the small intestine of pregnant mice by histological, enzyme histochemical, and immunohistochemical methods. Turkish Journal of Gastroenterology, 30, 917–924. 10.5152/tjg.2019.18681 PMC6812947 31625934

[phy270493-bib-0043] Soma‐Pillay, P. , Nelson‐Piercy, C. , Tolppanen, H. , & Mebazaa, A. (2016). Physiological changes in pregnancy. Cardiovascular Journal of Africa, 27, 89–94. 27213856 10.5830/CVJA-2016-021 PMC4928162

[phy270493-bib-0044] Teerapornpuntakit, J. , Klanchui, A. , Karoonuthaisiri, N. , Wongdee, K. , & Charoenphandhu, N. (2014). Expression of transcripts related to intestinal ion and nutrient absorption in pregnant and lactating rats as determined by custom‐designed cDNA microarray. Molecular and Cellular Biochemistry, 391, 103–116. 24519337 10.1007/s11010-014-1992-8

[phy270493-bib-0045] Volk, N. , & Lacy, B. (2017). Anatomy and physiology of the small bowel. Gastrointestinal Endoscopy Clinics of North America, 27, 1–13. 27908510 10.1016/j.giec.2016.08.001

