# Supplementary material for: Differential changes in anatomical, molecular and functional determinants of intestinal glucose absorption during murine pregnancy
Source: Physiol Rep. 2025 Aug 5;13(15):e70493. doi: 10.14814/phy2.70493 (PMC12325091; doi:10.14814/phy2.70493)
Supplement: Supplementary file 1 — Appendix S1. [file PHY2-13-e70493-s001.docx]

**Differential changes in anatomical, molecular and functional determinants of intestinal glucose absorption during murine pregnancy.**

T Sebastian Overduin^#1,3,4^, Georgia S Clarke^#1,3,4^, Hui Li^1,3^, Richard L Young^2,3^, Kathryn L Gatford^*1,3,4^, Amanda J Page^*1,3^

^1^School of Biomedicine and ^2^Adelaide Medical School, The University of Adelaide, SA 5000, Australia; ^3^Nutrition, Diabetes & Gut Health, Lifelong Health Theme, South Australian Health and Medical Research Institute, Adelaide, SA 5000, Australia; ^4^Robinson Research Institute, The University of Adelaide, Adelaide, Australia

^#, *^ Denotes equal contributions

^*^Co-senior and corresponding authors

**Supplementary Data**

The tables below report exact *P*-values for pairwise comparisons shown by letters on Figures in the main paper.

**Figure 1.**

**Panel B – villi length**

| **Pair-wise comparison between regions** | ***P*-value** |
| --- | --- |
| Duodenum cf. jejunum | < 0.001 |
| Duodenum cf. ileum | < 0.001 |
| Jejunum cf. ileum | < 0.001 |

**Panel C – crypt depth**

| **Pair-wise comparison between regions** | ***P*-value** |
| --- | --- |
| Duodenum cf. jejunum | 1.000 |
| Duodenum cf. ileum | < 0.001 |
| Jejunum cf. ileum | < 0.001 |

**Panel D – smooth muscle thickness**

| **Pair-wise comparison between regions** | ***P*-value** |
| --- | --- |
| Duodenum cf. jejunum | 0.033 |
| Duodenum cf. ileum | < 0.001 |
| Jejunum cf. ileum | < 0.001 |

**Figure 2.**

**Panel A: *Slc2a5*, P-values for comparisons between pregnancy stages within jejunum**

|  | **Early-pregnant** | **Mid-pregnant** | **Late-pregnant** |
| --- | --- | --- | --- |
| **Non-pregnant** | 1.000 | 0.012 | 1.000 |
| **Early-pregnant** | - | 0.197 | 1.000 |
| **Mid-pregnant** | - | - | 0.012 |
| **Late-pregnant** | - | - | - |

**Panel B: *Slc2a2*, P-values for comparisons between pregnancy stages within ileum**

|  | **Early-pregnant** | **Mid-pregnant** | **Late-pregnant** |
| --- | --- | --- | --- |
| **Non-pregnant** | 0.747 | 1.000 | 0.039 |
| **Early-pregnant** | - | 0.747 | 1.000 |
| **Mid-pregnant** | - | - | 0.109 |
| **Late-pregnant** | - | - | - |

**Panel C: *Slc5a1,*** **P-values for comparisons between pregnancy stages within ileum**

|  | **Early-pregnant** | **Mid-pregnant** | **Late-pregnant** |
| --- | --- | --- | --- |
| **Non-pregnant** | 0.211 | 1.000 | 0.004 |
| **Early-pregnant** | - | 0.555 | 0.738 |
| **Mid-pregnant** | - | - | 0.015 |
| **Late-pregnant** | - | - | - |

**Figure 3.**

**Panel A - *Slc6a19* expression**

| **Pair-wise comparison between regions** | ***P*-value** |
| --- | --- |
| Duodenum cf. jejunum | 0.001 |
| Duodenum cf. ileum | 0.107 |
| Jejunum cf. ileum | < 0.001 |

**P-values for comparisons between pregnancy stages**

|  | **Early-pregnant** | **Mid-pregnant** | **Late-pregnant** |
| --- | --- | --- | --- |
| **Non-pregnant** | 1.000 | 0.075 | 1.000 |
| **Early-pregnant** | - | 0.150 | 1.000 |
| **Mid-pregnant** | - | - | 0.006 |
| **Late-pregnant** | - | - | - |

**Panel B - *Slc6a6* expression**

| **Pair-wise comparison between regions** | ***P*-value** |
| --- | --- |
| Duodenum cf. jejunum | < 0.001 |
| Duodenum cf. ileum | 0.030 |
| Jejunum cf. ileum | 0.004 |

**Panel C - *Slc15a1* expression**

| **Pair-wise comparison between regions** | ***P*-value** |
| --- | --- |
| Duodenum cf. jejunum | 0.034 |
| Duodenum cf. ileum | 1.000 |
| Jejunum cf. ileum | 0.024 |

**Figure 4.**

**Panel A - *Cd36* expression**

| **Pair-wise comparison between regions** | ***P*-value** |
| --- | --- |
| Duodenum cf. jejunum | < 0.001 |
| Duodenum cf. ileum | 0.002 |
| Jejunum cf. ileum | < 0.001 |

**Panel B - *Fabp2* expression**

| **Pair-wise comparison between regions** | ***P*-value** |
| --- | --- |
| Duodenum cf. jejunum | < 0.001 |
| Duodenum cf. ileum | < 0.001 |
| Jejunum cf. ileum | < 0.001 |

**P-values for comparisons between pregnancy stages**

|  | **Early-pregnant** | **Mid-pregnant** | **Late-pregnant** |
| --- | --- | --- | --- |
| **Non-pregnant** | 0.010 | 0.032 | < 0.001 |
| **Early-pregnant** | - | 1.000 | 1.000 |
| **Mid-pregnant** | - | - | 0.728 |
| **Late-pregnant** | - | - | - |

**Figure 5.**

**Panel C – carbachol-induced Δ*I*_sc_**

**P-values for comparisons of between SI regions**

|  | **Distal Duodenum** | **Proximal Jejunum** | **Distal Jejunum** | **Proximal Ileum** | **Distal Ileum** |
| --- | --- | --- | --- | --- | --- |
| **Proximal Duodenum** | 1.00 | 0.621 | 0.048 | <0.001 | 0.004 |
| **Distal Duodenum** | - | 0.251 | 0.033 | <0.001 | 0.004 |
| **Proximal Jejunum** | - | - | 1.000 | 0.820 | 1.000 |
| **Distal Jejunum** | - | - | - | 1.000 | 1.000 |
| **Proximal Ileum** | - | - | - | - | 1.000 |
| **Distal Ileum** | - | - | - | - | - |
